# The small RNA RssR regulates *myo*-inositol degradation by *Salmonella enterica*

**DOI:** 10.1038/s41598-018-35784-8

**Published:** 2018-12-10

**Authors:** Carsten Kröger, Johannes E. Rothhardt, Dominik Brokatzky, Angela Felsl, Stefani C. Kary, Ralf Heermann, Thilo M. Fuchs

**Affiliations:** 1 Department of Microbiology, School of Genetics and Microbiology, Moyne Institute of Preventive Medicine, Trinity College, Dublin, Ireland; 2 Lehrstuhl für Mikrobielle Ökologie, ZIEL – Institute for Food & Health, Wissenschaftszentrum Weihenstephan, Technische Universität München, Weihenstephaner Berg 3, 85354 Freising, Germany; 3 Biozentrum, Bereich Mikrobiologie, Ludwig-Maximilians-Universität München, Großhaderner Str. 2-4, 82152 Martinsried/München, Germany; 4 Friedrich-Loeffler-Institut, Institut für molekulare Pathogenese, Naumburger Str. 96a, 07743 Jena, Germany

## Abstract

Small noncoding RNAs (sRNAs) with putative regulatory functions in gene expression have been identified in the enteropathogen *Salmonella enterica* serovar Typhimurium (*S*. Typhimurium). Two sRNAs are encoded by the genomic island GEI4417/4436 responsible for *myo*-inositol (MI) degradation, suggesting a role in the regulation of this metabolic pathway. We show that a lack of the sRNA STnc2160, termed RssR, results in a severe growth defect in minimal medium (MM) with MI. In contrast, the second sRNA STnc1740 was induced in the presence of glucose, and its overexpression slightly attenuated growth in the presence of MI. Constitutive expression of RssR led to an increased stability of the *reiD* mRNA, which encodes an activator of *iol* genes involved in MI utilization, via interaction with its 5′-UTR. SsrB, a response regulator contributing to the virulence properties of salmonellae, activated *rssR* transcription by binding the sRNA promoter. In addition, the absence of the RNA chaperone Hfq resulted in strongly decreased levels of RssR, attenuated *S*. Typhimurium growth with MI, and reduced expression of several *iol* genes required for MI degradation. Considered together, the extrinsic RssR allows fine regulation of cellular ReiD levels and thus of MI degradation by acting on the *reiD* mRNA stability.

## Introduction

*Salmonella enterica* serovar Typhimurium (*S*. Typhimurium) infects both animal and human hosts, and it is a major cause of diseases, including enteric fever, gastroenteritis, bacteraemia and systemic infection. *S*. Typhimurium is mainly transmitted by contaminated food, such as egg and its products, poultry, and pork. In mice, this pathogen evokes a disseminated infection that serves as a model for human typhoid fever. During infection, *S*. Typhimurium is challenged by various physical, biochemical, or cellular barriers such as low pH, bile, antimicrobial peptides, colonization resistance or phagocytes^1–3^. These stress conditions are overcome by specific virulence factors that have been characterized in detail, including those encoded by the *Salmonella* pathogenicity island 1 (SPI-1) or 2 (SPI-2) that are responsible for epithelial cell invasion, and survival and replication within non-phagocytic host cells or professional phagocytes^4–7^.

**Figure 1:**
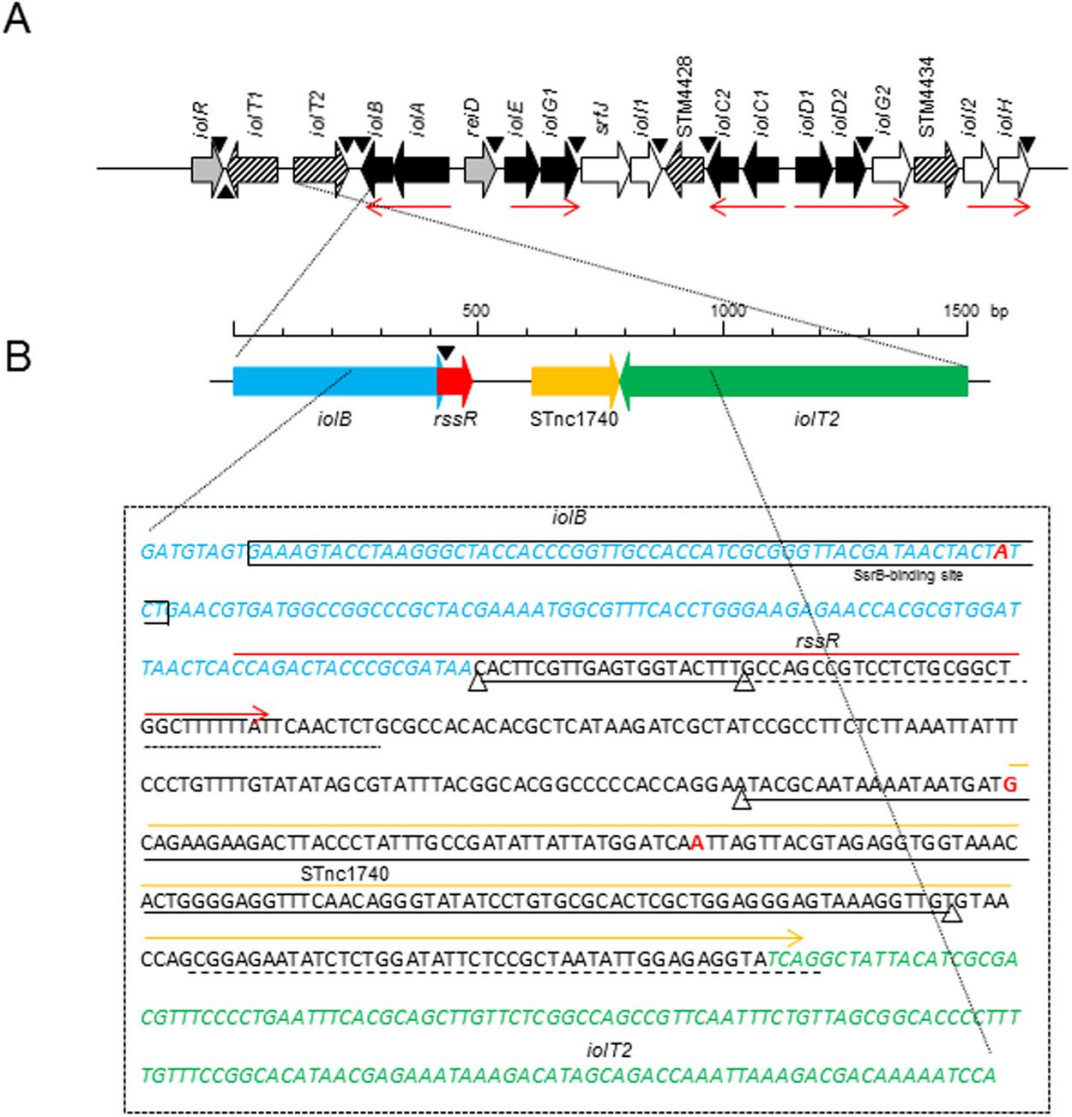
Identification of two small noncoding (sRNAs) within GEI4417/4436. (**A**) Genetic organization of GEI4417/4436. Genes essential for *myo*-inositol (MI) degradation are depicted in black, and the regulatory genes *iolR* and *reiD* in gray. Transporter genes are depicted in a hatched pattern. Red arrows indicate transcriptional units, and triangles indicate *luxCDABE* insertion sites used in the present study. (**B**) Chromosomal sequence spanning from *iolB* (nucleotide sequence in blue) to *iolT2* (nucleotide sequence in green). The sequences of the small RNA genes *rssR* and STnc1740 are marked by a red and a yellow line, respectively. Triangles connected by a black line mark the partial deletions of *rssR* and STnc1740 as described in the text. The SsrB binding region is shown as a square within *iolB* as identified by ChIP^38^. Nucleotides in red indicate potential TSS, and dashed lines depict ρ-independent terminators as predicted by the TransTermHP algorithm^79^; *iolB* and *rssR* share the same transcriptional terminator.

However, much less emphasis has been put on to the metabolic capacities of *S*. Typhimurium as a prerequisite for successful survival and proliferation in environments such as soil, food or host compartments that are characterized by variable or limited availability of nutrients^8–13^. An example of a metabolic pathway that facilitates recovery from nutrient deprivation is the capability of certain *S*. *enterica* strains to use *myo*-inositol (MI) as the sole carbon and energy source^14,15^. MI is a polyol abundant in soil and within body compartments of mammals including the bloodstream^16^, and it is an important building block for phosphatidylinositol and other membrane molecules of eukaryotes. The phosphorylated form of MI, inositol hexakisphosphate or phytate, serves as a phosphorus storage form in plants; however, this form can only be utilized by livestock in the presence of bacterial phytases. Species within the genera *Bacillus*, *Klebsiella*, *Corynebacterium*, *Clostridium*, *Lactobacillus*, *Rhizobium*, *Sinorhizobium*, and *Pseudomonas* are known to carry *iol* genes required for MI degradation, suggesting an origin of this specific metabolic property in soil bacteria^17,18^. In *S*. Typhimurium, the *iol* genes are located on a 22.6-kb genomic island (GEI4417/4436) (Fig. 1A). *In vivo* screening identified *iol* genes as candidate genes under selection during the oral infection of mice, pigs, chicken and calves^19–22^.

A unique feature of *S*. Typhimurium growth under standard laboratory conditions on minimal medium (MM) with MI is the long lag phase of approximately two days that can be strongly reduced by the deletion of *iolR*, whose product represses most *iol* genes. There is a strong selection pressure on a high binding affinity of IolR to its target promoters, because untimely expression of the *iol* genes during growth in rich medium and in the absence of IolR results in a high economic burden for *S*. Typhimurium^23^. The lag phase is also shortened by the addition of bicarbonate^17,24^; large amount of this electrolyte is secreted by the proximal duodenum^25,26^ and might serve as an *in vivo* signal to trigger MI degradation. During growth on solid MM medium with MI, strain 14028 exhibits a reversible bistable phenotype; however, this phenotype is absent in *iolR*-negative strains and in the presence of bicarbonate^24^. The phenotypic bistability is characterized by two subpopulations that consist of either proliferating or nongrowing cells. This phenomenon, which is accompagnied by a hysteresis effect, could be correlated with the activity of one of the *iol* promoters (P_*iolE*_) that in the presence of MI switches from the “off” to the “on” status by an as yet unknown mechanism^24,27^.

However, the complex regulation of the *iol* genes *in vitro* or *in vivo* is not yet fully understood. Recently, we identified ReiD, which is encoded by an orphan gene (STM4423) and is unique to *S*. Typhimurium strains capable of using MI. ReiD is a regulator that acts as a DNA-binding protein to induce the expression of  several *iol* genes, thus contributing to the regulation of MI degradation by *S*. Typhimurium^14^. ReiD stimulates the transcription of P_*iolE*_, the promoter of the *iolE*/*iolG1* (STM4424/STM4425) operon that encodes the first two enzymes of the MI degradation pathway. Gene *reiD* is also known to be significantly induced in a mouse enteritis model, but not in the typhoid fever mouse model^28^. Interestingly, mutants with a transposon insertion between the MI transporter gene *iolT2* (STM4419) and *iolB* (STM4420) encoding an isomerase involved in MI utilization, were found to be attenuated in pig, chicken and calf oral infection models^21^. RNA-sequencing (RNA-seq) and Hfq-Co-Immunoprecipitation (Co-IP) identified the presence of two small noncoding RNAs (sRNAs) within that intergenic region, suggesting that they might be responsible for the reported attenuation^29–31^. As many sRNAs are important and versatile regulatory elements that are involved in numerous cellular processes, including carbon metabolism and virulence in enteric bacteria^32,33^, we investigated the role of the GEI4417/4436-encoded sRNAs STnc1740 and RssR (STnc2160) in regulating MI catabolism. The present study shows that STnc1740 and RssR negatively and positively, respectively, influence the growth properties of *S*. Typhimurium using MI as carbon and energy source. We also provide strong experimental evidence that RssR interacts with and stabilizes the mRNA of *reiD*, and that its own transcription can be induced by the virulence regulator SsrB. The results suggest that RssR in particular contributes to the metabolic adaptation of *S*. Typhimurium under nutrient-limited conditions.

## Results

### Identification of two small RNAs located in GEI4417/4436

An RNA-seq-based transcriptomic analysis recently identified 280 sRNAs in *S*. Typhimurium strain 4/74. Among them are the two contiguous sRNAs STnc1740 and STnc2160 with a predicted length of 180 and 69 nucleotides, respectively (Fig. 1B)^29,30^. The sequences of both sRNAs are identical in the common laboratory strains LT-2, SL1344, 4/74, and 14028, and are encoded within the genomic island GEI4417/4436 that harbors the genes that are required for MI utilization. Both sRNAs are therefore proposed to play a role in the regulation of this metabolic capacity. When measured under 22 distinct *in vitro* growth conditions, STnc2160 was only strongly upregulated following anaerobic shock, whereas STnc1740 was expressed under most of the growth conditions^30^. STnc2160 is located in the 3′-untranslated region (3′-UTR) of *iolB* and partially overlaps with the coding region of *iolB*, whereas STnc1740 lies in the intergenic region between *iolB* and *iolT2* (Fig. 1B). Due to the experimental results outlined below, we termed STnc2160 as “RssR” for *reiD* mRNA-stabilizing small RNA. Its sequence is present (sequence identity 100%) in all the 46 *Salmonella* genome sequences that also carry *reiD*, but absent in the 23 genomes lacking this regulatory gene^14^.

### Presence of RssR and STnc1740 in mutant strains

**Figure 2:**
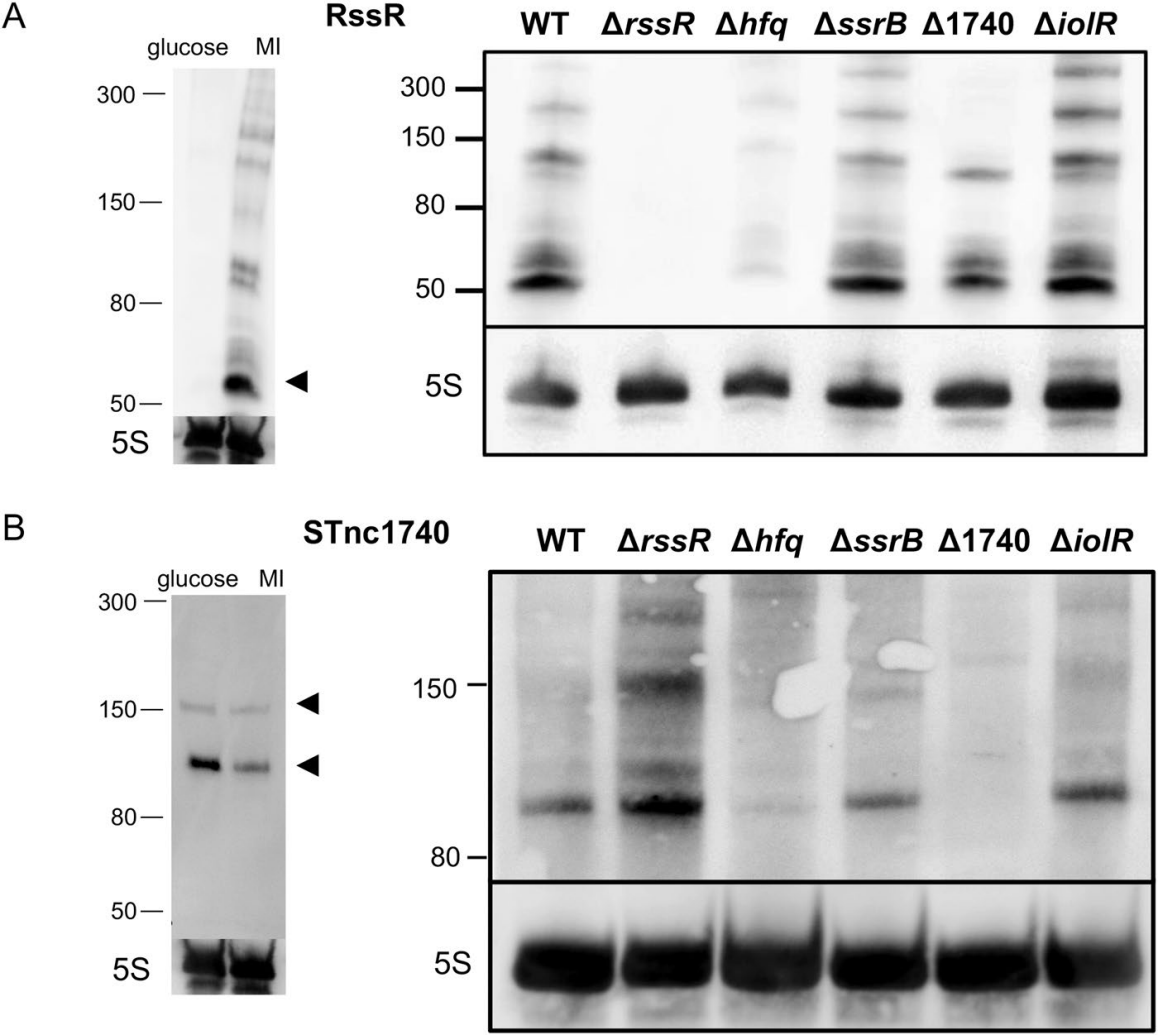
Northern blots to detect (**A**) RssR and (**B**) STnc1740 transcription. Left side: RNA was isolated from cells grown to OD_600_ = 0.3 in MM with 27.8 mM glucose or with 55.5 mM MI. Right side: RNA isolated from strain 14028 and its deletion mutants lacking *hfq*, *ssrB*, STnc1740, *iolR* and *rssR* grown in MI medium was used. Total RNA of 5 μg was loaded into each lane of a 7% urea-PAA gel, separated and blotted onto a nylon membrane. Single-strand digoxigenin (DIG)-labelled riboprobes were generated by *in vitro* transcription (RssR, riboprobe size: 62 nt; STnc1740, riboprobe size: 95 nt). 5S rRNA served as loading control. Arrowheads indicate most prominent bands corresponding to the TSS mentioned in the text.

To investigate the roles of RssR and STnc1740 in MI metabolism in strain 14028, we first studied the expressions of the two sRNAs in MM with MI or glucose as the sole carbon source. Northern blotting with a riboprobe complementary to *rssR* against total RNA isolated from *S*. Typhimurium 14028 cells grown to the exponential phase in MM with MI revealed a prominent hybridization signal with a size of ~70 nucleotides in MM with MI (Fig. 2A, left). This finding is in agreement with earlier data obtained from cells grown in rich medium until the early stationary phase^29^. Remarkably, RssR was highly expressed only in *S*. Typhimurium cells grown with MI, but not detected at all in the presence of glucose. Using the same RNA sample, we detected two distinct hybridization signals using a probe against STnc1740 (~150 and ~100 nucleotides) (Fig. 2B, left). This finding suggests the presence of two promoters for STnc1740 and is in agreement with two transcriptional start sites (TSS) that were identified by differential RNA-seq set apart by a 43-bp distance^30^. In clear contrast to the *rssR* transcript, the expression of the smaller transcript of STnc1740 was significantly higher in MM with glucose than in MM with MI, whereas the expression of the longer transcript was unaffected by the carbon source added.

Northern blots probing for *rssR* expression were then performed with RNA isolated from the mutants 14028 Δ*rssR*, 14028 Δ*hfq*, 14028 Δ*ssrB*, 14028 ΔSTnc1740 and 14028 Δ*iolR* grown in MI medium (Fig. 2A, right). We detected no hybridization signal in 14028 Δ*rssR* and only very low amounts of RssR in 14028 Δ*hfq* (see below), whereas RssR was present in the RNA isolated from strains 14028 Δ*ssrB*, 14028 ΔSTnc1740 and 14028 Δ*iolR*. RNA samples of the same strains were also tested with a STnc1740 probe, and this sRNA was found to be present in 14028 Δ*rssR*, 14028 Δ*ssrB*, and 14028 Δ*iolR*, but to be drastically reduced in mutant 14028 Δ*hfq* (see below) (Fig. 2B, right) and absent in 14028 ΔSTnc1740. We hypothesized that RssR, which is encoded by a gene located at the 3′-UTR region of *iolB*, can be generated either by transcription from its own promoter located within the coding region of *iolB* or by processing from the *iolB* mRNA via RNaseE^31,34^. To address this point, we used strain LT2 *rne*^TS^ (MA3409) in which the RNaseE is active at 28 °C, but not at 44 °C^35^. Strains LT2 (MA9816)and LT2 *rne*^TS^ were grown in MI medium at 28 °C until OD_600_ = 0.3 and then further incubated at 44 °C for one hour. The data shown in Fig. S1 demonstrate that in the strain with restricted RNase E activity, the number of fragments that hybridize with the RssR riboprobe is increased compared to the wild-type strain, indicating reduced RNA degradation in the *rne*^TS^ mutant of the *iolB* mRNA. Notably, the 60–70 nt RssR band visible in strain 14028 is much less pronounced in the *rne*^TS^ mutant, suggesting that RssR is processed from a longer transcript. However, our data do not distinguish whether mature RssR is processed from the *iolB* mRNA or from a longer precursor RssR transcript originating from within the *iolB* coding region, and a 5′RACE experiment was unsuccessful to confirm the predicted *rssR* transcriptional start site shown in Fig. 1.

### sRNAs influence the growth behavior of *S*. Typhimurium in *myo*-inositol medium

**Figure 3:**
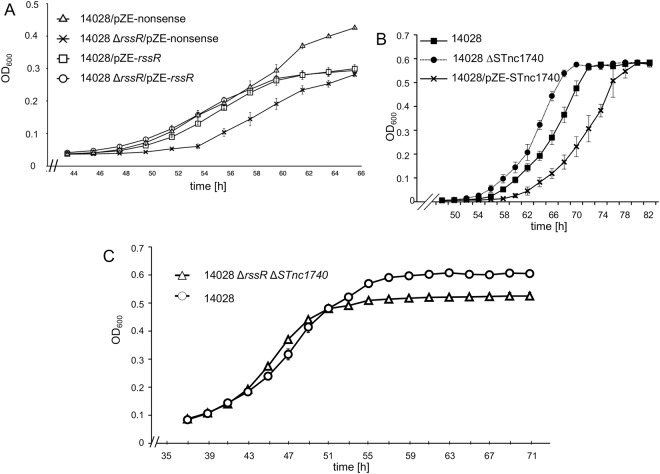
Growth phenotypes of sRNA deletion mutants of *S*. Typhimurium. Growth curves of (**A**) strains 14028, 14028 Δ*rssR*, 14028/pZE-*rssR* and 14028 Δ*rssR*/pZE-*rssR*, (**B**) strains 14028, 14028 ΔSTnc1740, and 14028/pZE-STnc1740, and (**C**) strain 14028 and the double mutant 14028 Δ*rssR* ΔSTnc1740. All strains were grown in MM with MI at 37 °C. Data points in all graphs represent mean values of three independent cultures; standard deviations are depicted.

We then tested a possible impact of RssR and STnc1740 on MI utilization by *S*. Typhimurium strain 14028. The doubling time of 14028 ∆*rssR* during growth in lysogeny broth (LB) medium did not significantly differ from that of the parental strain (Table S1). However, in MM with MI, the mutant showed a significantly (p ≤ 0.01) lower division rate [ν_(∆*rssR*)_ = 0.130 h^−1^ ± 0.030] in MM with MI compared to that of strain 14028 [ν = 0.310 h^−1^ ± 0.090], and a longer lag phase (Fig. 3A). In the case of 14028, the presence of plasmid pZE-control carrying a noncoding 17 bp-fragment (Table S2) resulted in a higher maximal optical density at 600 nm (OD_600_) in comparison with the other strains. When we constitutively expressed RssR in the mutant from the plasmid pZE-*rssR* to compensate for the lack of RssR, the division rate of this strain [ν_(∆*rssR*/pZE-*rssR*)_ = 0.261 h^−1^ ± 0.010] and the lag phase were restored to nearly that of strain 14028. A similar result was obtained for 14028 carrying the complementing construct [ν_(pZE-*rssR*)_ = 0.261 h^−1^ ± 0.017]. The successful complementation also suggests that the deletion of *rssR* does not significantly compromise *iolB* expression and function that is an essential gene for MI degradation. Taken together, these data indicate that RssR positively affects utilization of MI as the sole carbon and energy source.

In LB medium, the doubling times of 14028 ΔSTnc1740 and of 14028 ΔSTnc1740/pZE-STnc1740 with *in trans* expression of this sRNA were identical to that of parental strain 14028 (Table S1). During growth with MI, the doubling time of 14028 ΔSTnc1740 [t_d (∆STnc1740)_ = 3.01 h ± 11.6%] did not differ significantly from that of strain 14028 [t_d (14028)_ = 3.15 h ± 16.4%] (Fig. 3B), whereas that of strain 14028/pZE-STnc1740 overexpressing this sRNA [t_d (pZE-STnc1740)_ = 4.74 h ± 15.6%] is significantly increased in comparison with the other two strains (p < 0.001). In addition, the absence of STnc1740 shortens, and its *in trans* expression prolongs the lag phase during growth with MI. These data indicate that STnc1740, in contrast to RssR, inhibits the growth rate of *S*. Typhimurium 14028, although pleiotropic effects caused by STnc1740 overproduction cannot be excluded.

When testing the growth phenotype of the double mutant 14028 ∆*rssR* ΔSTnc1740, we observed a slightly but significantly higher division rate [ν_(Δ*rssR* ∆STnc1740)_ = 0.26 h ± 0.016%] compared to that of strain 14028 [ν_(14028)_ = 0.21 h ± 0.004%; p < 0.01] (Fig. 3C). Subsequently, we focused our investigation on RssR due to its more distinct effect on *S*. Typhimurium growth with MI.

### RssR increases *reiD* mRNA levels

**Figure 4:**
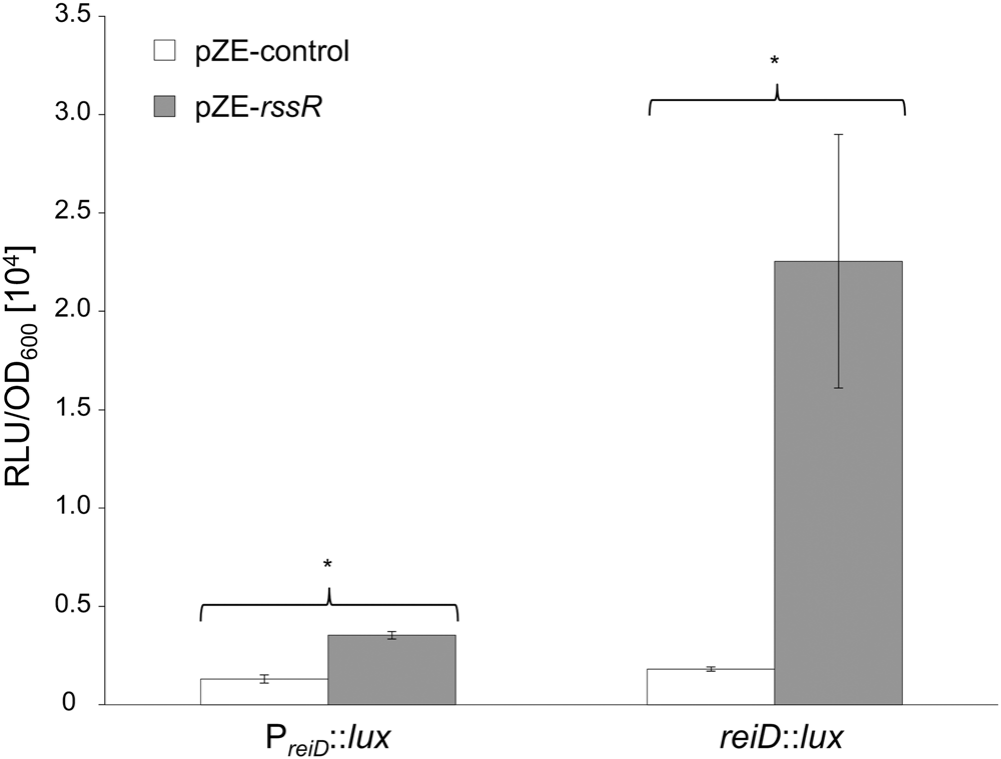
Role of RssR in the posttranscriptional regulation of *reiD*. Bioluminescence of the reporter strains 14028 P_*reiD*_::*lux* and 14028 *reiD*::*lux* harboring pZE-*rssR* was derived during growth in LB medium. Construct pZE-control carries a noncoding fragment of 14 nt (Table S2). The maximal transcriptional activities are shown as RLU/OD_600_. Each reporter experiment was independently performed in triplicate with three cultures each, and standard deviations are indicated. Significant differences (p < 0.05) are indicated by asterisks.

As shown above, deletion as well as constitutive *in trans* expression of RssR results in growth phenotypes of *S*. Typhimurium in MM with MI. This finding led to the assumption that RssR regulates an mRNA encoded on GEI4417/4436. To test this hypothesis, the luciferase reporter cassette *luxCDABE* was chromosomally fused to the end of each polycistronic *iol* operon or *iol* gene essential for MI degradation as determined previously^17^. The resulting strains (Table S2) were then equipped with plasmid pZE-*rssR* to allow constitutive expression of RssR. Changes of the luciferase activity of the constructs were not detected in strains with pZE-*rssR* in comparison with those with the control plasmid pZE-control (data not shown), except strain 14028 *reiD*::*lux*. Here a 12.4-fold increase of bioluminescence with respect to the control was observed (Fig. 4). Strain 14028 P_*reiD*_::*lux* harboring a *luxCDABE* fusion to the region upstream of the *reiD* start codon showed only a marginal, but significant signal increase. Due to these data, we hypothesize that RssR stabilizes the mRNA transcript of *reiD*.

### Deletion of *rssR* destabilizes the mRNA of *reiD*

**Figure 5:**
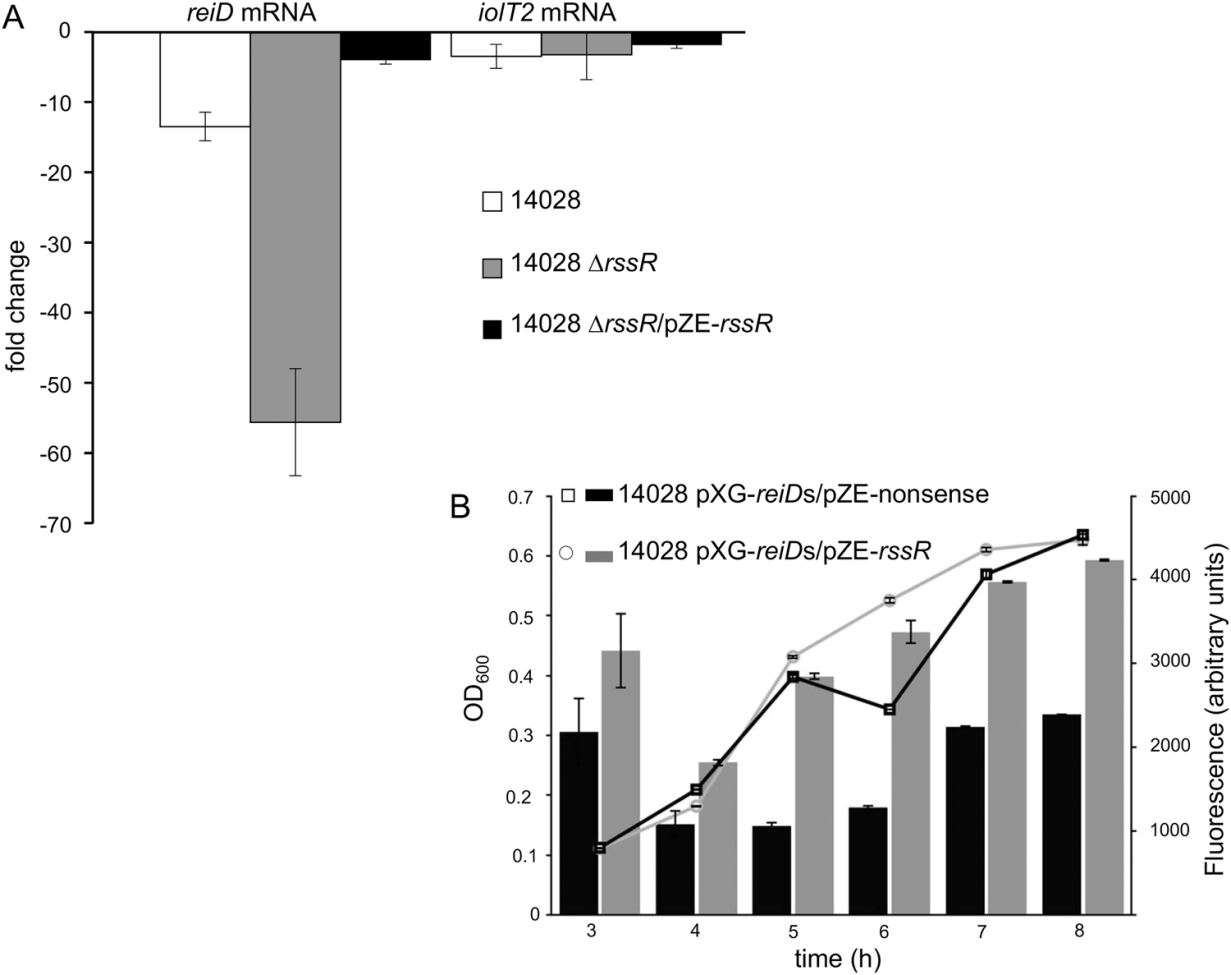
The stability of the *reiD* mRNA is affected by RssR. (**A**) Strains 14028, 14028 Δ*rssR* and 14028 Δ*rssR*/pZE-*rssR* were cultivated in liquid MI medium, and rifampicin was added to a final concentration of 500 µg/mL at OD_600_ 0.3. Subsequently, mRNA was isolated from cultures 2 min before and 8 min after transcriptional inhibition. The transcription of *reiD* and *iolT2* was quantified by qRT-PCR, and the fold changes of transcripts in relation to 16S rRNA were calculated. The arithmetic averages and standard deviations derived from three independent experiments performed in duplicate are shown. (**B**) Strains 14028 pXG-*reiD*s/pZE-nonsense and 14028 pXG-*reiD*s/pZE-*rssR* were grown in LB medium until the stationary phase. GFP-production is indicated in arbitrary fluorescence units. The mean values (±standard deviation) of three independent experiments are shown.

To further investigate the putative function of RssR in stabilizing the mRNA of *reiD*, strains 14028 and 14028 ∆*rssR* were cultivated in MM with MI to an OD of 0.3, and transcription was halted by adding 500 µg/mL rifampicin. Quantitative real-time PCR (qRT-PCR) against *reiD*, and as a control, *iolT2* transcripts, was performed, and the data were normalized to the 16S rRNA detection level. Comparing the values obtained for time points 2 min before and 8 min after the transcriptional stop, we calculated a 13.5-fold decay of the *reiD* mRNA isolated from 14028 within these 10 min. However, the *reiD* mRNA obtained from mutant 14028 ∆*rssR* showed a 55.6-fold reduction (p ≤ 0.01), clearly suggesting that RssR indeed slows down the degradation of the *reiD* mRNA, indicating a specific effect of RssR on the *reiD* transcript stability (Fig. 5A). We then complemented deletion strain 14028 ∆*rssR* with the plasmid pZE-*rssR*, and detected an only 4.05-fold reduction of the *reiD* mRNA amount. Thus, the constitutive *in trans* expression of RssR compensated the chromosomal lack of *rssR* and led to a significantly higher stability of the *reiD* mRNA in strain 14028 ∆*rssR*/pZE-*rssR* in comparison with the deletion mutant (p < 0.01). No significant difference between the samples of the three strains was observed when qRT-PCR against the transcript of the control gene *iolT2* was performed, excluding that the overexpression or deletion of RssR affects the stability of cellular RNA in general.

### Interaction of RssR and the *reiD* mRNA

We then applied the two-plasmid-system pXG-10(sf) and pZE12-luc (Table S2) to measure the stability of the *reiD* mRNA in the presence of RssR via green fluorescent protein (GFP) production^36,37^. The UTR 5′-sequence and the coding region of gene *reiD* were cloned into pXG-10(sf), resulting in a translational coupling of *gfp* to *reiD*; the recombinant protein was controlled by the constitutive P_LtetO_ promoter. The *rssR* gene was cloned into pZE12-luc downstream of the constitutive P_LlacO_ promoter. Following transformation of both plasmids into strain 14028, the fluorescence was measured during growth in LB medium for 8 h until the cells reached the stationary phase. A significant higher fluorescence of strain 14028/pXG-*reiD*/pZE-*rssR* in comparison with strain 14028/pXG-*reiD*/pZE-control suggested a stabilizing function of RssR for the *reiD* mRNA (data not shown). To narrow the sequence relevant for interaction, we cloned the first 150 bp following the TSS of *reiD* into pXG-10(sf), resulting in pXG-*reiD*short (pXG-*reiD*s). Again, we observed a significantly, up to 2.67-fold higher fluorescence relative to the control (Fig. 5B).

**Figure 6:**
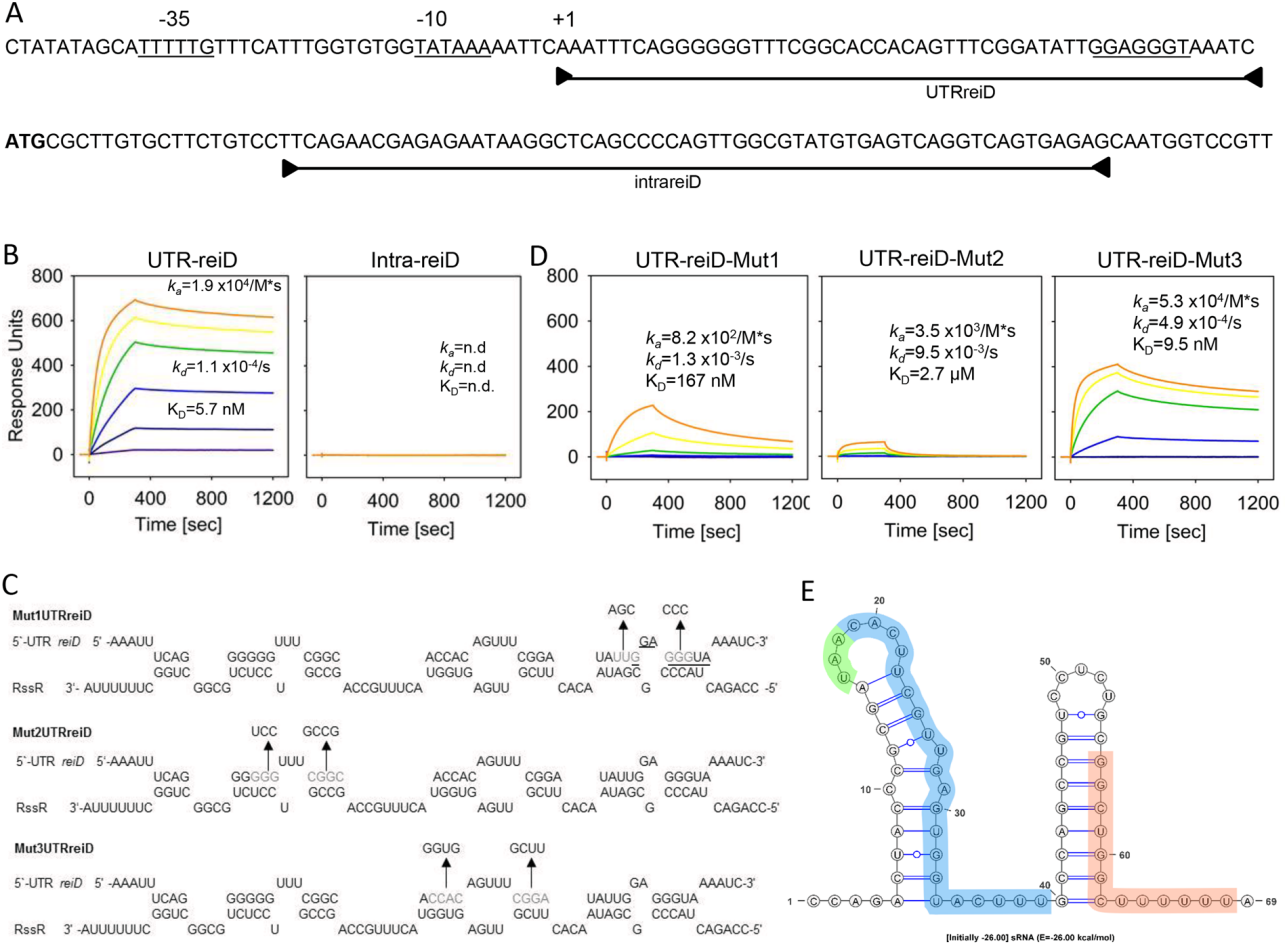
SPR spectroscopy of *reiD*-binding to RssR. (**A**) The 5′-UTR of *reiD* and the first 92 nucleotides of its coding region. The TSS and the −10 and −35 consensus sequences are indicated, as well as the fragments from which the RNA oligonucleotides used for SPR spectroscopy were derived. The Shine-Dalgarno sequence is underlined. The biotin-labeled sRNA RssR was captured on a streptavidin-coated sensor chip, and purified RNA oligonucleotides UTRreiD and intrareiD were passed over the chip at a flow rate of 30 µl/min and temperature of 25 °C (concentrations of 0, 10, 50, 100, 250, 500, 1000 nM) using a contact (association) time of 180 sec, followed by a 900-sec dissociation phase. The resulting sensorgrams are shown in (**B**). The binding properties of the mutant oligonucleotides Mut1UTRreiD, Mut2UTRreiD, and Mut3UTRreiD (**C**) were also qualified *via* Biacore (**D**). (**E**) Secondary structure of RssR as predicted by mfold^80^ and visualized using VARNA^81^. In blue: nucleotides missing in mutant 14028 Δ*rssR*; in green: *iolB* stop codon; in red: binding site of *hfq*^45^. A free energy of −26.0 kcal/mol was calculated.

To further validate the RssR-*reiD* interaction, we performed a binding  kinetic analysis via surface plasmon resonance (SPR) spectroscopy. Biotinylated  RssR was bound on a sensor chip and tested with two RNA-oligonucleotides representing the 5′-UTR of *reiD* (UTRreiD) and the nucleotides 20 to 80 of the *reiD* coding region (intrareiD)(Fig. 6A). Oligonucleotide UTRreiD was demonstrated to specifically and stably interact with RssR with an overall affinity of 5.7 nM and a high association (1.9 × 10^4^/M*s) and low dissociation rate (1.1 × 10^−4^/s), whereas no binding was detected with oligonucleotide intrareiD (Fig. 6B).

To identify the 5′-UTR nucleotides most relevant for the interaction with RssR, a *reiD* 5′-UTR/RssR duplex structure was predicted, and at least seven potential binding regions between the two RNA-molecules were found. Six of them were pairwise mutated (Fig. 6C), and the resulting RNA-oligonucleotides Mut1UTRreiD, Mut2UTRreiD and Mut3UTRreiD were tested for binding to RssR via SPR spectroscopy. RssR showed a binding affinity to Mut3UTRreiD similar to that to the parental sequences (9.5 nM), although the maximal binding response was two-fold reduced (Fig. 6D). In contrast, the interaction strength of RssR with Mut1UTRreiD was strongly reduced (167 nM) due to lower association (8.2 × 10^2^/M*s) and lower dissociation constants (1.3 × 10^−3^/s). Furthermore, the maximal binding response was approximately four-fold reduced. Only weak binding of RssR to Mut2UTRreiD was observed, with an overall affinity of 2.7 µM. The binding stoichiometries of a least 4:1 UTRreiD to RssR, of 2:1 for Mut3UTRreiD and of 1:1 for Mut2UTRreiD, depended on the maximal response of the respective sensorgrams, might be due to a different self-binding or oligomerization of the different UTRreiD derivatives or caused by a different number of binding sites on RssR for the respective UTRreiD derivative. The putative secondary structure of RssR is shown in Fig. 6E. Taken together, these data strongly suggest that RssR stabilizes the mRNA of *reiD* by direct interaction. We hypothesize that the nucleotide mismatches in Mut2UTRreiD predominately contribute to this interaction, because the binding sites tested with Mut1UTRreiD belong to the putative Shine-Dalgarno sequence of *reiD*.

### SsrB binds and induces expression of P_*rssR*_

**Figure 7:**
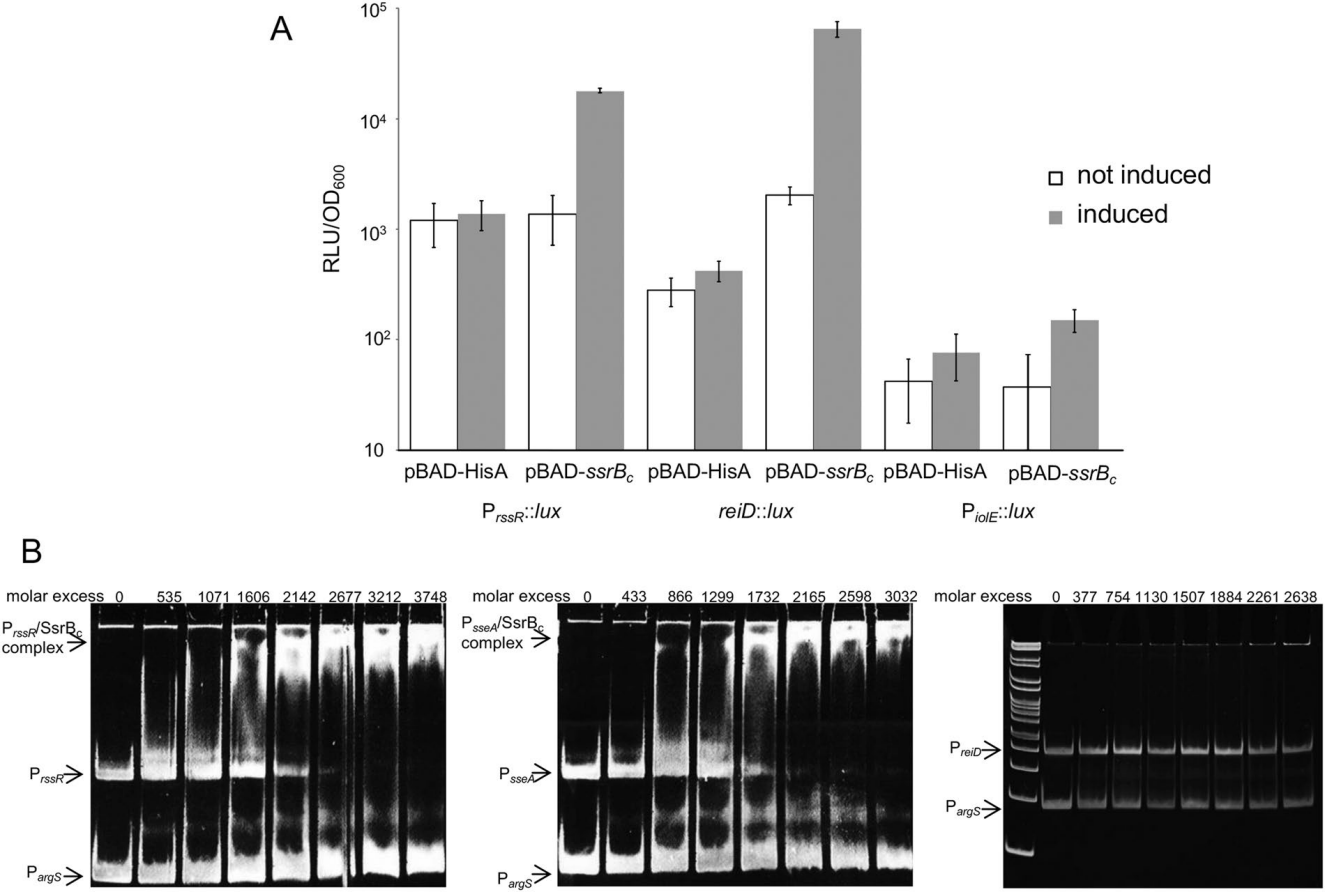
Interaction of SsrB_c_ with the promoter of *rssR*. (**A**) Reporter strain 14028 P_*rssR*_::*lux* carrying pBAD-HisA(Tet^R^) or pBAD-*ssrB*_*c*_ was grown in LB medium in the absence and presence of 1 mM arabinose. The maximal transcriptional activities measured as RLUs were normalized to the OD_600_ (RLU/OD_600_). Standard deviations of three independently performed experiments with three cultures each are shown. (**B**) GMSAs with purified SsrBc against fragments representing the promoters of *rssR* (left), *sseA* (middle), or *reiD* (right) were performed with 12% native polyacrylamide gels. The promoter of *argS* served as a negative and competitive control in each experiment. Arrows indicate protein/DNA complexes, and the molar excess of protein over DNA is depicted above each lane.

A genome wide ChIP-on-chip approach identified an intragenic SsrB binding site that is located 70 bp upstream of *rssR* and thus within the coding region of *iolB*^38^, and analysis with a promoter prediction program^39^ accordingly found evidence for a TSS immediately behind this region (Fig. 1B). This finding prompted us to validate a putative interaction of SsrB with the promoter of *rssR* (P_*rssR*_), and we constructed pBAD-*ssrB*_*c*_ encoding the C-terminus of *ssrB*. SsrB_c_ was chosen here, as this domain is constitutively active and binds DNA without conformational activation by SsrA (SpiR)^40^. As a positive control, the reporter strain 14028 *sseA*::*lux* carrying a chromosomal fusion of the luciferase reporter with *sseA* was equipped with pBAD-*ssrB*_*c*_. The luminescence activity in strain 14028 *sseA*::*lux*/pBAD-*ssrB*_*c*_ increased from 6.6 × 10^3^ relative light units (RLU)/OD_600_ [±5.9%] in the absence of inducer, to 6.4 × 10^6^ RLU/OD_600_ [±1.4%] in the presence of 1 mM arabinose, demonstrating the functionality of SsrB_c_. Reporter strains 14028 P_*rssR*_::*lux*, 14028 *reiD*::*lux*, and 14028 P_*iolE*_::*lux* were then transformed with pBAD-*ssrB*_*c*_ and, as a control, with plasmid pBAD-HisA(Tet^R^) lacking *ssrB*_*c*_, and bioluminescence measurements were performed in the absence and the presence of arabinose. Figure 7A shows that the transcriptional activity of P_*rssR*_::*lux* and *reiD*::*lux* was approximately 13-fold and 155-fold induced following SsrB_c_ overproduction, respectively, whereas such an effect was not observed in the absence of arabinose or with control strain 14028/pBAD-HisA(Tet^R^) nor with the P_*iolE*_-reporter strain.

To validate this finding, SsrB_c_ was overexpressed from pBAD-*ssrB*_*c*_ in *Escherichia coli* KB3, purified and used for gel mobility shift assays (GMSAs). The promoter of *sseA* served as positive control and that of *argS* as a competitive DNA^17,41^. The GMSAs shown in Fig. 7B demonstrate that SsrB_c_ binds P_*rssR*_ at approximately the same molar ratio as the positive control P_*sseA*_. The specificity of this interaction was further demonstrated by an additional bandshift experiment in which SsrB_c_ failed to bind the promoter of the regulatory gene *reiD*. Equal amounts of RssR were detected in a Northern blot performed with RNA samples isolated from 14028 and its *ssrB* deletion mutant grown in MM/MI (Fig. 2B), suggesting that SsrB stimulates *rssR* transcription under distinct, SsrB-inducing conditions, for example those encountered during infection or biofilm formation^42,43^. Considered together, we conclude that activated SsrB can specifically bind P_*rssR*_ and induces transcription of *rssR*, but is not essential for the expression of RssR in MM with MI.

### Deletion of *hfq* results in a severe growth defect of *S*. Typhimurium in MI medium

**Figure 8:**
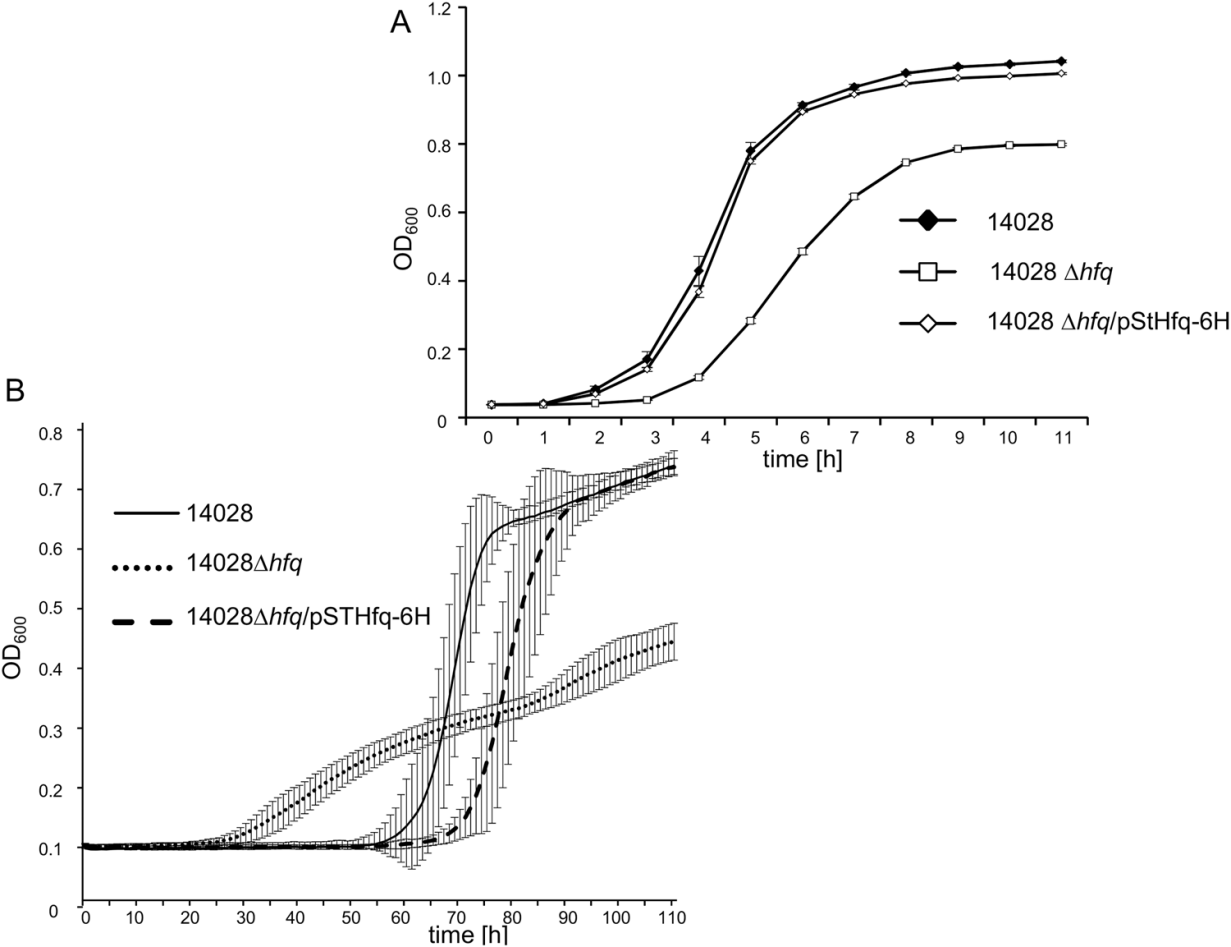
Influence of Hfq on growth phenotypes. Strains 14028 and 14028 Δ*hfq* were grown in flasks at 37 °C in LB broth (**A**) or in microtiter plates in MM with MI (**B**). Experiments were performed in triplicate, and average data from ten wells per experiment as well as the standard deviations are shown.

The RNA chaperone Hfq is known to affect the stability of sRNAs and their annealing with mRNAs^44^. In contrast to STnc1740, RssR is strongly bound by the RNA chaperone Hfq at position 56–68 including its terminator^45^. Accordingly, the lack of RssR in the *hfq*-mutant suggests an sRNA-stabilizing interaction of RssR and Hfq (Fig. 1B). The association of RssR with Hfq suggests that this sRNA posttranscriptionally regulates the expression of mRNA targets potentially transcribed from GEI4417/4436. To investigate the influence of Hfq on the MI metabolism of *S*. Typhimurium, the deletion mutant 14028 ∆*hfq* was constructed, and its growth behavior was monitored. In LB broth, the *hfq* minus strain exhibited a slightly reduced growth rate as compared to the parental strain [t_d (14028)_ = 0.91 h ± 6.7%; t_d (14028∆*hfq*)_ = 0.97 h ± 2.2%], and a lag phase prolonged by a few hours (Fig. 8A). Strain 14028 ∆*hfq* also showed a weaker total growth as it reached a maximal OD_600_ ~ 0.8 in comparison with OD_600_ ~ 1.0 measured for 14028, probably due to a pleiotropic effect of this mutation. The phenotype of the parental strain was restored by providing plasmid pStHfq-6H.

However, during growth with MI, we observed a more severe growth attenuation of the *hfq* mutant that showed a significantly longer doubling time [t_d(14028∆*hfq*)_ = 8.85 h ± 3.2%] as compared to that of strain 14028 [t_d (14028)_ = 3.87 h ± 3.4%] (Fig. 8B). In these experiments, which were performed in microtiter plates, the final OD_600_ of the mutant was also reduced from OD_600_ ~ 0.60 to OD_600_ ~ 0.38. Moreover, the lag phase of 14028 (55 h) was strongly reduced to approximately 37 h by deletion of *hfq*. The deletion of *hfq* was successfully complemented by plasmid pStHfq-6H, as growth of 14028 ∆*hfq*/pStHfq-6H was very similar to that of the parental strain (Fig. 8B). The lack of RssR in strain 14028 ∆*hfq* during growth in MM with MI (Fig. 2B) further suggests that the growth impairment of this mutant is due to a reduced expression and/or stability of RssR. This is in agreement with RNA-seq results showing a 4.3-fold down-regulation of RssR in a *S*. Typhimurium strain 4/74 Δ*hfq* mutant grown in LB medium to the early stationary phase^46^.

**Figure 9:**
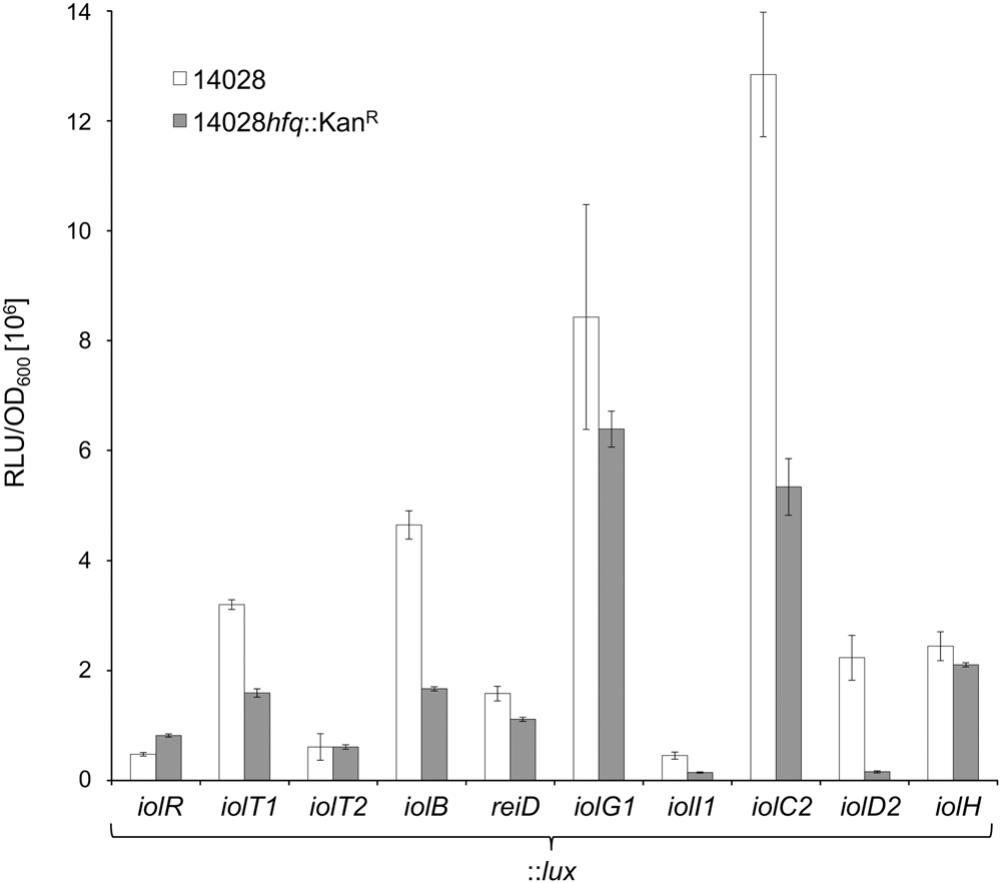
Transcriptional analysis of *iol* genes in 14028 and mutant 14028 *hfq*::Kan^R^ by chromosomal *luxCDABE* reporter fusions during growth in MM with MI. Data points are mean values of three independent cultures represented by three wells each; standard deviations are depicted.

To shed further light on the role of Hfq in the regulation of the MI degradation pathway, we fused the luciferase reporter behind 10 *iol* genes or operons within strain 14028 ∆*hfq* and monitored their bioluminescence profile in comparison with that of the corresponding fusions in strain 14028 during growth in MM with MI. Remarkably, the luciferase activity of all but two translational fusions significantly decreased by *hfq* deletion (Fig. 9). The exceptions were *iolT2*::*lux* encoding a minor inositol transporter^47^ with equal transcription in both strains, and *iolR*::*lux* with slightly elevated activity. The strongest response, namely an approximately 14-fold decrease of transcriptional activity, was observed for the *iolD2*::*lux* fusion, and even the transcription of the MI-transporter gene *iolT1* showed a twofold reduction. A negative effect of the *hfq* deletion on construct *reiD*::*lux* was also observed. Taken together, these data are compatible with the fact that Hfq interacts with RssR that then stabilizes the mRNA of *reiD* whose product is the main activator of *iol* genes^14^.

## Discussion

It is generally accepted that bacterial sRNAs are regulators of gene expression and perform a broad range of physiological functions. In contrast to the *cis*-encoded antisense RNAs, *trans*-encoded sRNAs typically range from 50 to 300 nucleotides, and exhibit only imperfect complementarity with their RNA target^48,49^. Two modes of action by which those noncoding RNAs modulate gene expression are most common. One class of sRNAs can directly interact with a protein to modify its activity^50,51^, whereas the other base-pairs imperfectly in an Hfq-dependent manner with cognate mRNA targets and thus inhibits initiation by masking the ribosomal binding site followed by mRNA destabilization via RNAse E, or liberate a sequestered RBS, a mechanism termed anti-antisense that results in translational activation^44,52^. In *S*. Typhimurium, sRNAs play important roles in regulating virulence and metabolic properties^32^. Examples of the latter category are the control of amino acid metabolism including the branched chain amino acids via GcvB^53^, the role of SgrS in glucose homeostasis^54–57^, and uptake of chitin-derived oligosaccharides involving ChiX^35^.

Recently, a detailed transcriptome analysis of *S*. Typhimurium growing in a set of environmental, stress or gut mimicking conditions revealed the expression of 280 sRNAs^30^. However, the functional characterization of many remains incomplete. Here, we present the characterization of a sRNA termed RssR that is involved in the regulation of the MI degradation pathway in *S*. Typhimurium. We found that the sRNA RssR, whose gene *rssR* overlaps with the coding region and the 3′-UTR of *iolB*, probably interacts with and stabilizes the mRNA of *reiD via* interaction with the 5′-UTR, thus controlling the expression of this regulatory gene at the posttranscriptional level. Examples of sRNAs that activate gene expression upon interaction with a target mRNA by stemloop formation and via the 5′-UTR have been described^52,58^. However, we do not exclude the possibility that the phenotypes observed in this study are the indirect results from translation stimulation of *reiD* by sequestration of an anti-Shine Dalgarno sequence^59^. In comparison with a strain lacking RssR, the presence of RssR results in a higher abundance of ReiD mRNA. The regulator then induces the genes *iolE*/*iolG1* that are essential for MI degradation and encode the first enzymes of this pathway. Therefore, RssR positively regulates MI utilization by targeting *reiD* and promotes growth of *S*. Typhimurium in environments with MI as a carbon and energy source. Although the deletion of sRNA STnc1740 has a less prominent growth effect in comparison with that of RssR, its growth phenotype indicates that STnc1740 counteracts the effect of RssR by a yet unknown mechanism.

There is increasing evidence that the capacity to degrade MI might contribute to the survival, colonization and growth of *S*. Typhimurium in several hosts^14,19–21,28,60^. Interestingly, a transposon-directed insertion-site sequencing (TraDIS) application identified a transposon mutation in *rssR* to attenuate *S*. Typhimurium growth following oral infection of calves, chickens, and pigs^21^. The long lag phase of *S*. Typhimurium during growth with MI might place doubt on the possible relevance of MI utilization during infection. However, the tight regulation of this metabolic pathway can in part be overcome by bicarbonate, which is present in the gastrointestinal tract as demonstrated recently^24^. Alternatively, as hypothesized here, the MI metabolism might be supported by the common virulence regulator SsrB. SsrB has also been identified to induce *srfJ* that is located on the MI degradation island^41,61,62^. Together with the sensor SsrA (SpiR), the response regulator SsrB forms a two-component system that is responsible for the induction of the SPI-2 located type III secretion system and effector proteins essential for survival in macrophages^63^. In our study, we confirm the observation that SsrB binds to a site within GEI4417/4436^38^, namely the *rssR* promoter, and demonstrate that SsrB can activate the novel sRNA RssR, but is not required for RssR expression in medium with MI as sole carbon and energy source. SsrB induction has been linked to expression in macrophages and recently in its unphosphorylated form to biofilm formation^43^, whereas MI utilization is not induced inside macrophages^64,65^. However, recent studies show that the SsrB-regulated SPI-2 genes are already expressed in the gut lumen^66,67^ where the expression of *iol* genes might additionally be favored by the presence of bicarbonate. Our data suggest that mature RssR is produced by processing of the *iolB* mRNA or of an RssR precursor RNA that is transcribed from its own promoter(s) within the *iolB* coding region by SsrB and/or other regulatory factors, or both. As SsrB binding to a site within *iolB* might impair transcription of this gene, we hypothesize an only temporary interaction to stimulate the activation of the MI degradation pathway.

The expressions of almost a fifth of all *S*. Typhimurium genes are controlled by the RNA-binding protein Hfq that facilitates the efficient stabilization and annealing of small, regulatory RNAs to their cognate mRNA targets upon direct interaction^31,44,49,68^. The Hfq regulon not only includes genes involved in pathogenicity or the flagellar cascade, but also those involved in fatty acid biosynthesis, in the metabolism of amino acids, nitrogen, purine and pyrimidine, or sugar uptake and utilization^68^. The postulated binding of RssR by Hfq^31^ prompted us to study the effect of an *hfq* mutant on MI degradation. We demonstrate that in a rich medium, strain 14028 ∆*hfq* showed a weaker total growth than strain 14028, as similarly observed previously in minimal acidic medium, indicating that Hfq controls the regulation of growth rate^69^. However, such a pleiotropic effect was not observed with a *hfq* deletion mutant of strain SL1344^70^, a distinction that might be strain-specific or due to the growth conditions. More intriguingly, a lack of Hfq resulted in a severe growth defect of *S*. Typhimurium in MM with MI, and we hypothesize that the overall reduced transcription of most *iol* genes in the *hfq* mutant contributes to this phenotype, although pleiotropic effects of the Hfq deletion cannot be excluded. Similar to a *iolR* deletion, a lack of Hfq reduces the lag phase in the presence of MI by many hours, suggesting that Hfq acts on the cellular levels of IolR. Lower amounts of IolR result in an earlier expression of catabolic *iol* genes, thus shortening the lag phase in MI medium. Deletion of Hfq under non-inducing conditions of the *iol* genes, such as early stationary phase or LB medium, was recently shown to up-regulate catabolic *iol* genes^46^, pointing to a yet unknown, additional regulatory mechanism that fine-regulates the IolR repressor. The co-immunoprecipitation results with Hfq^31^ and the reduction of the cellular level of RssR by the *hfq* deletion indicate that Hfq binds and thus stabilizes RssR. In parenthesis, a contrary finding has recently been reported for Hfq of *Yersinia enterocolitica* that represses the utilization of several substrates including MI^71^.

Taken together, we identified the positive contribution of sRNA RssR to the regulation of the MI utilization pathway by stabilization of the mRNA of the activator ReiD. RssR is probably an extrinsic RNA as it regulates the translation of a non-overlapping gene. Our data also suggest that the virulence regulator SsrB may control MI degradation *via* increasing the abundance of RssR, and thus of *reiD* mRNA, and possibly triggers the activation of this metabolic pathway during infection of the gastrointestinal tract. Together with hydrogen carbonate, a gut compound that reduces the lag phase of the MI utilization pathway, this regulatory mechanism allows a timely response to changing conditions. The findings presented here support the important role of RssR in fine-regulating MI degradation by *S*. Typhimurium.

## Methods

### Bacterial strains, plasmids and growth conditions

The bacterial strains and plasmids used in the present study are listed in Table S2. *S*. Typhimurium and *E*. *coli* cultures were grown in liquid or solid LB medium (10 g/L tryptone, 5 g/L yeast extract, 5 g/L NaCl) or MM [M9 medium supplemented with 2 mM MgSO_4_, 0.1 mM CaCl_2_ and 55.5 mM (1% wt/vol) MI or 27.8 mM (0.5% wt/vol) glucose]. For plasmid maintenance, the different media were supplemented with the following antibiotics: ampicillin (150 μg/mL), kanamycin (50 µg/mL), tetracycline (12 µg/mL) or chloramphenicol (20 µg/mL). For solid media, 1.5% agar (w/v) was added. For all growth and promoter probe experiments, bacterial strains were grown in appropriate medium overnight at 37 °C and then diluted 1:1,000 in liquid growth medium. Growth curves were derived from bacterial cultures incubated at 37 °C in 250 mL flasks with 50 mL medium or in 100-well plates using Bioscreen C (iLF bioserve, Langenau, Germany). The OD_600_ was measured at different time intervals as indicated. An amount of 1 mM (0.2% wt/vol) L(+) arabinose was used to stimulate the expression of genes cloned in pBAD/HisA(Tet^R^).

### Standard procedures

The manipulation and isolation of chromosomal or plasmid DNA were performed according to standard protocols^72^ and following the manufacturers’ instructions. Vector cloning was performed with *E*. *coli* strain TOP10. Plasmid DNA was transformed *via* electroporation using a Bio-Rad Gene pulser II as recommended by the manufacturer and as described previously^73^. PCRs were conducted using Taq polymerase (Fermentas, St. Leon-Rot, Germany). As a template for PCR, chromosomal DNA, plasmid DNA, or cells from a single colony were used. The oligonucleotides synthesized for PCRs are listed in Table S3. *S*. Typhimurium gene numbers refer to the LT2 annotation (NC 003197). The student’s t-test was applied for statistical evaluations. The RNAhybrid tool was applied for the *in silico* prediction of sRNA/RNA hybridization^74^.

### Construction of deletion mutants and recombinant plasmids

Deletion mutants of STnc1740, *rssR* and *hfq* (STM5242) were constructed using the λ-Red recombinase^75^. Briefly, PCR products containing the kanamycin resistance cassette of plasmid pKD4 and the flanking FRT sites were generated using primers of 70 nucleotides in length that included 20 nucleotides priming sequences for pKD4 as template DNA. The fragments were transformed into *S*. Typhimurium strain 14028 cells harboring plasmid pKD46, and the allelic replacement of the target gene was controlled by PCR. Nonpolar deletion mutants were obtained by transformation of pCP20, and were validated by PCR analysis and DNA sequencing. The sequences of STnc1740 and *hfq* were precisely deleted, whereas in the case of mutant 14028 Δ*rssR*, the first 18 nucleotides of *rssR* overlapping with *iolB* remained in the chromosome.

For constitutive expression, sRNAs were cloned using the pZE12-*luc* plasmid (Expressys, Ruelzheim, Germany) according to^36^. For that purpose, part of pZE12-*luc* was amplified using the primers PLlacoB and PLlacoD and treated with XbaI, resulting in two fragments of ~2.2 kb and a 1.7 kb fragment. The longer fragment containing the vector backbone, was then ligated with XbaI-restricted STnc1740 or *rssR* fragments amplified from the 14028 chromosome using the primers listed in Table S3, resulting in plasmids pZE-STnc1740 and pZE-*rssR*. To overproduce a highly active variant of SsrB termed SsrB_c_^40^ with N-terminal His_6_-tag, nucleotides 412–639 of *ssrB* were cloned into pBAD/HisA(Tet^R^). Recombinant pXG10(sf) plasmids were constructed via SLIC^76^. All plasmids were verified by PCR and sequencing. Enzymes (Fermentas) used are listed in Table S2 and Table S3.

### Cloning of promoter fusion to *luxCDABE*

To construct chromosomal reporter strains, 500 bp-fragments representing the region upstream of the start codon (promoters) or the 3′-region of a gene or operon were amplified from *S*. Typhimurium 14028 DNA by PCR using the primers listed in Table S3. The fragments were then cloned upstream of the promoterless *luxCDABE* genes into the multiple cloning site of the suicide vector pUTs-*lux*(Cm^R^). After transformation into *E*. *coli* SM10 cells, plasmids were validated by PCR and sequencing. The constructs were transferred into 14028 or derivatives by conjugation, and exconjugants were selected and verified by PCR. Enzymes (Fermentas) used are listed in Table S2 and Table S3.

### RNA isolation, quantitative real-Time PCR, and Northern blotting

Total RNA was isolated from *S*. Typhimurium 14028 and derivatives as follows: at appropriate time points, culture samples were taken and resuspended in TRIzol reagent (Sigma-Aldrich, Taufkirchen, Germany). RNA was then isolated as previously described^29^ and treated with DNaseI (Fermentas) twice to eliminate any DNA contamination. Synthesis of cDNA and qRT-PCR were performed as previously described^77^. Northern blotting was performed according to Kröger and colleagues^29^ using the DIG Northern blot starter kit (Roche, Penzberg, Germany) following the manufacturer’s manual; the RiboRuler High Range RNA ladder (Thermo Fisher, Waltham, MA, USA) was used as a marker. The oligonucleotides used for the amplification of non-radiolabeled riboprobes are listed in Table S3.

### Quantification of transcriptional activities

Bioluminescence measurements were performed according to the method by Rothhardt *et al*.^14^. For measurements in LB medium, cells were grown overnight at 37 °C and diluted 1:1,000 in LB medium. Samples of 200 µL were then analysed during incubation in a 96-well plate at 37 °C under shaking. To induce pBAD/HisA(Tet^R^)-derived overexpression, cultures were supplemented with 1 mM arabinose. The values shown in the figures represent the maximal transcriptional activity observed during the exponential growth phase.

### Purification of SsrB_c_

His_6_-SsrB_c_ was overproduced in *E*. *coli* BL21λDE3 lacking H-NS and the H-NS-like factor StpA from pBAD-*ssrB*_c_, and purified using the Ni-NTA Fast Start Kit (Qiagen, Hilden, Germany) as follows: an overnight culture of *E*. *coli* was diluted 1:100 into 400 mL LB medium and incubated at 37 °C and 180 rpm. After 3 h, the expression of *ssrB*_*c*_ was induced by adding 1 mM arabinose. Following a further incubation of 4 h, the cells were harvested, and the pellets were resuspended in 4 mL of native lysis buffer. The cells were lysed by ultrasonification (Sonopuls UW2200, Bandelin, Berlin), and the cell debris were removed by centrifugation at 4 °C (20 min, 1.6 × 10^4^ g) and filtration via Millex-GV (Merck, Cork, Ireland). His_6_-SsrB_c_ was bound to the column that was then washed and eluted according to the manufacturer’s protocol. The protein concentration was determined using RotiQuant solution (Carl Roth GmbH, Karlsruhe, Germany) based on the method of Bradford^78^. The purity of eluted fractions was analyzed by separation on a 12.5% sodium dodecyl sufate (SDS) polyacrylamide gel and Western blot according to^77^, revealing a ~10 kD protein.

### GMSAs with purified SsrB_c_

Putative promoter regions of *rssR*, *sseA*, and *argS* as competitor DNA, were amplified (for oligonucleotides, see Table S3 or^17^, and 100 ng of DNA was mixed with increasing amounts of purified His_6_-SsrB_c_ in 1 × Tris/borate/ ethylenediaminetetraacetic acid (EDTA) buffer (TBE) with a total volume of 20 µL. After incubation for 45 min at room temperature, the samples were loaded with 4 µL of 6 × loading dye (Fermentas) on a 12% native polyacrylamide gel prepared in 1 × TBE buffer and separated at 120 V for 3 h in the same buffer. DNA was then stained in ethidium bromide solution and visualized by ultraviolet (UV) irradiation.

### SPR spectroscopy

SPR spectroscopy assays were performed using a Biacore T200 device (GE Healthcare) and streptavidin-precoated Xantec SAD500-L carboxymethyl dextran sensor chips (XanTec Bioanalytics GmbH, Düsseldorf, Germany). Before immobilizing the DNA fragments, the chips were equilibrated by three injections using 1 M NaCl/50 mM NaOH at a flow rate of 10 µl min^−1^. Then, 10 nM of the RssR oligonucleotide labelled with cyanine at its 5′-end and with biotin-TEG at is 3′-end was injected using a contact time of 420 sec and a flow rate of 10 µl min^−1^ to a final response of 1000–5000 RU. As a final wash step, 1 M NaCl/50 mM NaOH/50% (v/v) isopropanol was injected. Then, RNA oligonucleotides were injected over the surface for 180 s contact time following a dissociation tome of 900 s at flow rate 30 µl/min. After each cycle, bound RNA was removed from the chip by injecting 40% formamide, 3.6 M urea, and 30 mM EDTA for 120 s. All experiments were conducted at 25 °C with RNA structure buffer [100 mM Tris/HCl pH 7.0; 1 M KCl; 100 mM MgCl_2_]. Before use, all RNA molecules were denaturated for 5 min at 100 °C and renaturated by slowly cooling down the temperature to 25 °C. Sensorgrams were recorded using the Biacore T200 Control software 2.0 and analyzed with the Biacore T200 Evaluation software 2.0. The surface of flow cell 1 was not immobilized with RNA and used to obtain blank sensorgrams for subtraction of bulk refractive index background. The referenced sensorgrams were normalized to a baseline of 0.

## Electronic supplementary material

Supplementary Dataset 1
