# Supplementary material for: The small RNA RssR regulates myo-inositol degradation by Salmonella enterica
Source: Sci Rep. 2018 Dec 10;8:17739. doi: 10.1038/s41598-018-35784-8 (PMC6288124; doi:10.1038/s41598-018-35784-8)
Supplement: Supplementary file 1 — Supplementary Dataset 1 [file 41598_2018_35784_MOESM1_ESM.pdf]

Supplementary Information

The small RNA RssR regulates *myo*-inositol degradation by *Salmonella enterica*

Carsten Kröger[1#], Johannes E. Rothhardt[2#], Dominik Brokatzky[2], Angela Felsl[2], Stefani C. Kary[1], and Thilo M. Fuchs*[2, 3]

[1]Department of Microbiology, School of Genetics and Microbiology, Moyne Institute of Preventive Medicine, Trinity College, Dublin 2, Ireland; [2]Lehrstuhl für Mikrobielle Ökologie, ZIEL – Institute for Food & Health, Wissenschaftszentrum Weihenstephan, Technische Universität München, Weihenstephaner Berg 3, 85354 Freising, Germany; [3]Friedrich-Loeffler-Institut, Institut für molekulare Pathogenese, Naumburger Str. 96a, 07743 Jena, Germany

Running title: *myo*-inositol metabolism of *Salmonella typhimurium*

Keywords: *myo*-inositol, sRNA, regulation, metabolism, *Salmonella typhimurium*

[#]Both authors contributed equally to this study.

*Correspondence and requests for materials should be addressed to T.M.F. (email: thilom.fuchs@fli.de)

Supplementary Fig. S1. Northern blot to detect RssR in strain 14028 and its RNase E termosensitive mutant lgrown in MI medium. RNA was isolated from cells grown to OD_{600}=0.3 at 28°C and then shifted for one hour to 44°C. Total RNA of 5 µg was loaded into each lane of a 7% urea-PAA gel, separated and blotted onto a nylon membrane. A single-strand digoxigenin (DIG)-labelled riboprobe of RssR (size: 62 nt) was generated by *in vitro* transcription. 5S rRNA served as loading control. MA3409, derivative of *S.* Typhimurium LT-2 lacking Gifsy-1.

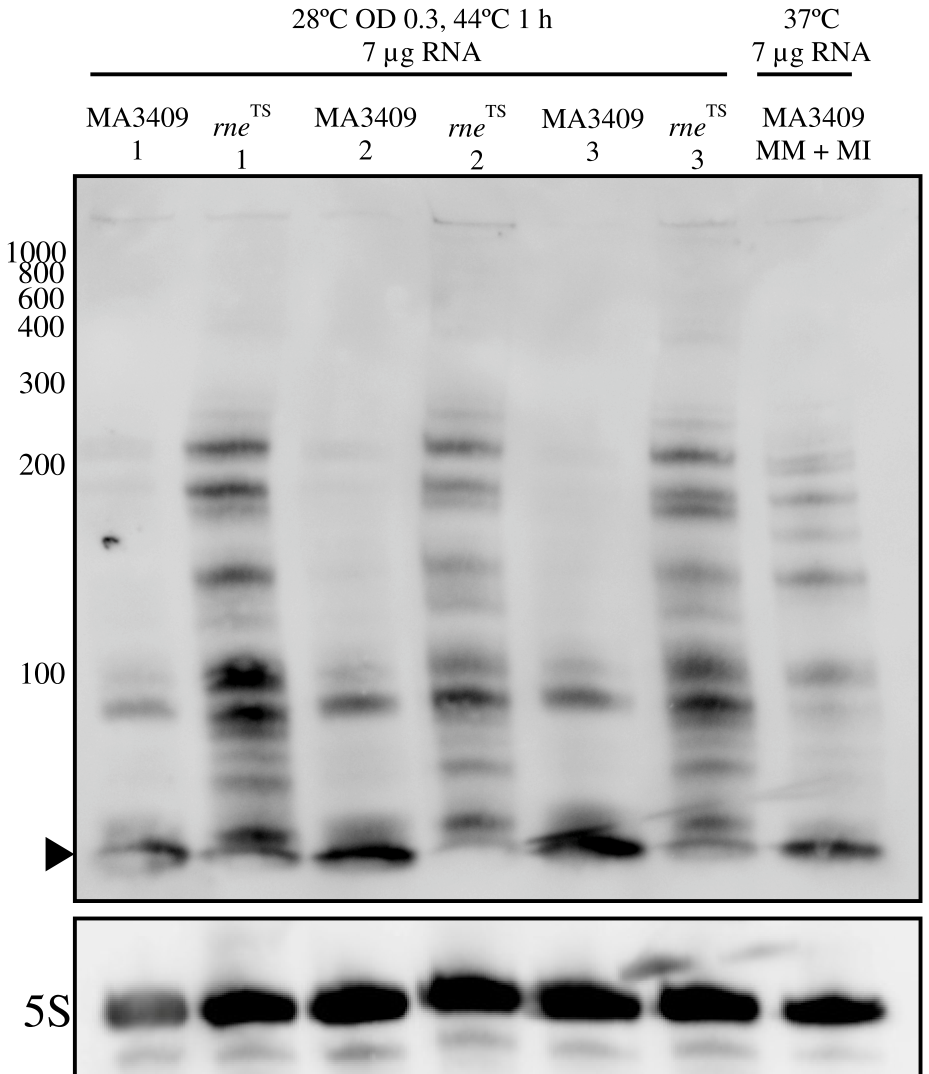

Table S1. Doubling time (t_d) of 14028, its sRNA mutants and complemented strains in LB medium.

Strain	doubling time t_d [h]	SD [%]	p-value*
14028	1.15	1.65	
14028Δ*rssR*	1.17	0.20	0.2005
14028Δ*rssR*/pZE-*rssR*	1,10	2.32	0.1057
14028Δ*STnc1740*	1.13	4.45	0.6897
14028Δ*STnc1740*/pZE-*STnc1740*	1.12	2.49	0.25503

* in relation to 14028

Table S2. Strains and plasmids used in this study.

**Bacterial strains**	**Description and relevant features**	**Source or literature**
E. coli		
SM10 λ*pir*	*lacY tonA recA* Mu_c+ *thi thr leu supE* RP4-2-Tc::Mu Km^R λ*pir*	[1]
TOP10	*F^-, mcrA, Δ(mcr-hsdRMS-mcrBC)Φ80lacZΔM15 ΔlacX74 recA1 araD139 Δ(ara-leu)7679, galU, galK, rpsL (Str^r), endA1, nupG*	Invitrogen, Karlsruhe, Germany
BL21λDE3 Δ*stpA*Δ*hns*	BL21λDE3 with a deletion of *stpA* and *hns*	[2]
***S.* Typhimurium**		
14028	*S.* Typhimurium strain ATCC14028, spontaneous Str^R mutant	[3]
14028 ΔSTnc1740	Partial STnc1740 deletion mutant in which the 3′-end representing the *iolT2* terminator was left in the chromosome to ensure termination of *iolT2* transcription	This study
14028 Δ*rssR*	Partial *rssR* (STnc2160) deletion mutant in which nucleotides of *rssR* overlapping with the *iolB* codon region and its putative terminator remained in the chromosome	This study
14028 Δ*hfq*	*hfq* deletion mutant	This study
LT2	MA3409	[4]
LT2 rne^{TS}	MA9816 (*rne*-3071), temperature-sensitive mutant of *rne*	[5]
14028 P_{rssR}::*lux*	Chromosomal fusion of the luciferase reporter to the *rssR* promoter	This study
14028 P_{iolE}::*lux*	Chromosomal fusion of the luciferase reporter to the *iolE* promoter	This study
14028 *sseA*::*lux*	Chromosomal fusion of the luciferase reporter immediately downstream of *sseA*	[6]
14028 P_{reiD}::*lux*	Chromosomal fusion of the luciferase reporter to the *reiD* promoter	[6]
14028 *iolX*::*lux*	Chromosomal fusion of the luciferase reporter immediately downstream of *iolR*, *iolT1*, *iolT2 iolB*, *reiD*, *iolG1*, *iolI1*, *iolC2, iolD2* and *iolH*	[6]

**Plasmids**		
pKD4	*pir*-dependent, FRT sites; Kan^R	CGSC,Yale [7]
pKD46	λ-Red helper plasmid; Amp^R	CGSC,Yale [7]
pCP20	FLP recombinase plasmid; Cm^R Amp^R	CGSC,Yale [7]
pUTs-*lux*(Cm^R)	Suicide plasmid derived from pUT mini-Tn*5* *luxCDABE* Km2 [8] without transposon, and with Kan^R substituted by Cm^R	[9]
pUTs-P$_{rssR}$::*lux*	pUTs-*lux*(Cm^R) with 500 bp *rssR* promoter fragment fused with *lux via* SacI/KpnI	This study
pBAD/HisA(Tet^R)	pBAD/HisA derived, arabinose-inducible expression vector with N-terminal 6xHis-tag; Tet^R	[9]
pBAD-*ssrB*$_C$	3´-end of *ssrB* encoding the 137 C-terminal amino acids cloned into pBAD/HisA(Tet^R) *via* NcoI/KpnI	This study
pZE12-*luc*	Vector carrying colE1 and constitutively active P$_{LlacO-1}$; Amp^R	Expressys, Ruelzheim, Germany; [10]
pZE-control	Non-coding fragment GCCTTAAGGTCTAG cloned into pZE-12-*luc*	This study
pZE-STnc1740	STnc1740 cloned into pZE12-*luc*	This study
pZE-*rssR*	Gene *rssR* cloned into pZE12-*luc*	This study
pXG-10(sf)	Vector with constitutively active P$_{LtetO-1}$ and superfolder *gfp*; Cam^R	[11]
pXG-*reiD*	UTR 5´-sequence and *reiD* cloned into pXG-10(sf)	This study
pXG-*reiD*(s)	First 150 bp after the TSS of *reiD* cloned into pXG-10(sf)	This study
pStHfq-6H	Gene *hfq* with own promoter and His$_6$-tag cloned into pZE12-*luc* derived pJV300; Amp^R	[12]

1 Simon, R., Priefer, U. & Pühler, A. A Broad Host Range Mobilization System for *in vivo* Genetic Engineering: Transposon Mutagenesis in Gram Negative Bacteria. *Biotechnology* **1**, 784-791 (1983).

2 Böhme, K. *et al.* Concerted actions of a thermo-labile regulator and a unique intergenic RNA thermosensor control *Yersinia* virulence. *PLoS Pathog* **8**, e1002518 (2012).

3 Kröger, C. & Fuchs, T. M. Characterization of the *myo*-inositol utilization island of *Salmonella enterica* serovar Typhimurium. *J Bacteriol* **191**, 545-554 (2009).
4 Figueroa-Bossi, N., Coissac, E., Netter, P. & Bossi, L. Unsuspected prophage-like elements in *Salmonella typhimurium*. *Mol Microbiol* **25**, 161-173 (1997).
5 Figueroa-Bossi, N., Valentini, M., Malleret, L., Fiorini, F. & Bossi, L. Caught at its own game: regulatory small RNA inactivated by an inducible transcript mimicking its target. *Genes & Dev* **23**, 2004-2015, doi:10.1101/gad.541609 (2009).
6 Rothhardt, J. E., Kröger, C., Broadley, S. P. & Fuchs, T. M. The orphan regulator ReiD of *Salmonella enterica* is essential for *myo*-inositol utilization. *Mol Microbiol* **94**, 700-712 (2014).
7 Datsenko, K. A. & Wanner, B. L. One-step inactivation of chromosomal genes in *Escherichia coli* K-12 using PCR products. *Proc Natl Acad Sci U S A* **97**, 6640-6645 (2000).
8 Winson, M. K. *et al.* Engineering the *luxCDABE* genes from *Photorhabdus luminescens* to provide a bioluminescent reporter for constitutive and promoter probe plasmids and mini-Tn*5* constructs. *FEMS microbiology letters* **163**, 193-202 (1998).
9 Starke, M., Richter, M. & Fuchs, T. M. The insecticidal toxin genes of *Yersinia enterocolitica* are activated by the thermolabile LTTR-like regulator TcaR2 at low temperatures *Mol Microbiol* **89**, 596-611 (2013).
10 Lutz, R. & Bujard, H. Independent and tight regulation of transcriptional units in *Escherichia coli* via the LacR/O, the TetR/O and AraC/I1-I2 regulatory elements. *Nucleic Acids Res* **25**, 1203-1210 (1997).
11 Corcoran, C. P. *et al.* Superfolder GFP reporters validate diverse new mRNA targets of the classic porin regulator, MicF RNA. *Mol Microbiol* **84**, 428-445, doi:10.1111/j.1365-2958.2012.08031.x (2012).
12 Sittka, A., Pfeiffer, V., Tedin, K. & Vogel, J. The RNA chaperone Hfq is essential for the virulence of *Salmonella typhimurium*. *Mol Microbiol* **63**, 193-217, doi:10.1111/j.1365-2958.2006.05489.x (2007).

Table S3. Oligonucleotides used in this study.

primer name	target gene	modification	5' – 3' sequence
construction of non-polar deletion mutants			
del_STnc1740_for	STnc1740		AGCGTATTTACGGCACGGCCCCCACCAGGAATACGCAATAAAATAATGATGTGTAGGCTGGAGCTGCTTC
del_STnc1740_rev			GAGATATTCTCCGCTGGTTACACAACCTTTACTCCCTCCAGCGAGTGCGCCATATGAATATCCTCCTTA
del_STnc2160_for	STnc2160		CCTGGGAAGAGAACCACGCGTGGATTAACTCACCAGACTACCCGCGATAAGTGTAGGCTGGAGCTGCTTC
del_STnc2160_rev			TAAGAGAAGGCGGATAGCGATCTTATGAGCGTGTGTGGCGCAGAGTTGAACATATGAATATCCTCCTTA
del_hfq_for	*STM5242* (*hfq*)		TTTCAGAATCGAAAGGTTCAAAGTACAAATAAGCATATAAGGAAAAGAGAGTGTAGGCTGGAGCTGCTTC
del_hfq_rev			AGCGGGGGCGATTATCCGACGCCCCCGACATGGATAAACAGCGCGTGAACCATATGAATATCCTCCTTA
test of insertion of kanR and gene deletion			
test_STnc1740_for	*STnc1740*		GGGTGCCGCTAACAGAAAT
test_STnc1740_rev			GTACTTTGCCAGCCGTC
test_STnc2160_for	*STnc2160*		TCGGCAAATAGGGTAAGTC
test_STnc2160_rev			TGTATGGCGGTCTATAACC
test_hfq_for	*STM5242* (*hfq*)		GGTGCTATCGCAGAC
test_hfq_rev			TGCTCACCGGCATCA
kanR3	*kanR*		GCGCTGCGAATCGGG
construction of pZE12-luc plasmids			
PLlacoB	*pZE-luc*		CGCACTGACCGAATTCATTAA
PLlacoD			GTGCTCAGTATCTTGTTATCCG
pZE12luc_seq_for	*pZE-luc* sequencing		GCACATTTCCCCGAAAAGTG

pZE12luc_seq_rev			CTGTGGATAACCGTATTACC
com_STnc1740_for	STnc1740		p-GCAGAAGAAGACTTACCCTA
com_STnc1740_rev		*XbaI*	GCGATCTAGAACAGGATATACCCTGTTGAA
com_STnc2160_for	STnc2160		p-CCAGACTACCCGCGATAACA
com_STnc2160_rev		*Xba*I	GCGATCTAGATAAAAAAGCCAGCCGCAGAG
com_nonsense_for			p-GCCTTAAGGT
com_nonsense_rev			CTAGACCTTAAGGC
construction of recombinant pBADHisA plasmids			
HISSSRBC5	*STM1391* (*ssrB*)	*Nco*I	CCTAGAACCATGGGACACCACCACCACCACCACGCTGAATTAAACGCTGACACG (Feng 2004)
pu_SsrBc_rev		*Kpn*I	GGGGTACCCCTTAATACTCTATTAACC
cloning, testing and sequencing of reporter fusions pUTs-luxCDABE			
PSTnc2160_for	$P_{STnc2160}$	*Sac*I	GCAGAGCTCTGGCCGTATGCAGCG
PSTnc2160_rev		*Kpn*I	CGGGGTACCTTATCGCGGGTAGTC
PSTnc2160_test			TCGGCAAATAGGGTAAGTC
reiD_for	*reiD*	*SacI*	GTGGAAAAACTGATGAGCTCCGTG
reiD_rev		*KpnI*	CGGGGTACCCAAATAATAAATAATTAGC
reiD_test			TCGTCCCGATGCTTTTCC
PiolE_for	P_{iolE}	*SacI*	GCAGAGCTCGAATTTAAACGCCGC
PiolE_rev		*KpnI*	CGGGGTACCCCACTTAATGAAACGC
PiolE_test			TCGTCCCGATGCTTTTCC
iolH_for	*iolH*	*SacI*	GCAGAGCTCAATTGCTGCCGATCGTGGA
iolH_rev		*KpnI*	CGGGGTACCTTAACGTCCGAGTAGCTC

iolH_test			GGTATTTACAATGAAAATTGC
sseA_for	*sseA*	*SacI*	GACGAGCTCGTAGTACGTGAGGTTTG
sseA_rev		*KpnI*	CGGGGTACCCCGTTACCTTTTTGTTTTTCCTG
sseA_test			GATCCGCAGCAATATCAG
pUTs_for	pUTs sequencing		GCAATTTTGAGTGACACAGG
luxC_rev	*luxC*		ACAACATCATAAAGGCCG
construction of pXG10(sf)-plasmids			
pXG10(sf)_for	pXG10(sf)		GCTAGCGGATCCGCTGGCTCCGCTGC
pXG10(sf)_rev			ATGCATGTGCTCAGTATCTCTATCAC
SLIC reiD_for			GTGATAGAGATACTGAGCACATGCATAACGCATACCGTATTCAATG
SLIC reiD1_rev	TSS_{reiD}+ *reiD*		GCAGCGGAGCCAGCGGATCCGCTAGCATATTCAAATAATAAATAATTAGC
SLIC reiD2_rev	TSS_{reiD} +150 bp *reiD*		GCAGCGGAGCCAGCGGATCCGCTAGCTCCTTCGGTGATAAGAATCA
pXG10(sf)_seq_for	pXG10(sf) sequencing		TAGGCGTATCACGAGGCC
pXG10(sf)_seq_rev			GGGACAACTCCAGTGAAAAG
construction of fragments used in GMS assay			
PargS_for	*STM1909* (*argS*)		CAACCTTTGATTTGATTGG
PargS_rev			AAGAGCCTGAATATTCAC
PSTnc2160_for	$P_{STnc2160}$	*Sac*I	GCAGAGCTCTGGCCGTATGCAGCG
PSTnc2160_rev		*Kpn*I	CGGGGTACCTTATCGCGGGTAGTC
PsseA_for	*STM1397* (*sseA*)		CGATGAGCTCACGGCAAGTTACAGGATCC
PsseA_rev			GCCGGTACCTCCCCTCCATATACACGATAG
PreiD_for	*STM4423* (*reiD*)		GGAATTCTGAAAGTTTGACGGCTG

PreiD_rev	GGGATCCCATACGCCAACTGGGGCTGA
quantitative real-time PCR	
qRT_16S_for	GTCTGTCAAGTCGGATGTG
qRT_16S_rev	AGATCTCTACGCATTTCACC
qRT_reiD_for	GATGATATTGTGCCGGTTAT
qRT_reiD_rev	ACTACCTGGGTCAATAGCTG
qRT_iolT2_for	ATCTACAGTGGATTAACGGC
qRT_iolT2_rev	ACCAACAGAATATAGCGGAG
Northern blot	
RP_STnc2160_for	CCAGACTACCCGCGATAAC
RP_STnc2160_rev	GAATTAATACGACTCACTATAGCCAGCCGCAGAGGACG
RP_STnc1740_for	GAAGACTTACCCTATTTGCCG
RP_STnc1740_rev	GAATTAATACGACTCACTATAGCACAGGATATACCCTGTTG
RP_5S_for	GCGGCACTAGCGCGGTGGTC
RP_5S_rev	GAATTAATACGACTCACTATAGCATGGGGAGACCCCACACT
SPR spectroscopy	
RssR	CCAGACUACCCGCGAUAACACUUCGUUGAGUGGUACUUUGCCAGCCGUCCUCUGCGGCUGGCUUUUUUA
intrareiD	UUCAGAACGAGAGAAUAAGGCUCAGCCCCAGUUGGCGUAUGUGAGUCAGGUCAGUGAGAG
UTRreiD	AAAUUUCAGGGGGGUUUCGGCACCACAGUUUCGGAUAUUGGAGGGUAAAUC
Mut1UTRreiD	AAAUUUCAGGGGGGUUUCGGCACCACAGUUUCGGAUAAGCGACCCUAAAUC
Mut2UTRreiD	AAAUUUCAGGGUCCUUUGCCGACCACAGUUUCGGAUAUUGGAGGGUAAAUC
Mut3UTRreiD	AAAUUUCAGGGGGGUUUCGGCAGGUGAGUUUGCUUUAUUGGAGGGUAAAUC

Supplementary information-uncropped Northern blots from Fig. 2 Kröger *et al*. 2018

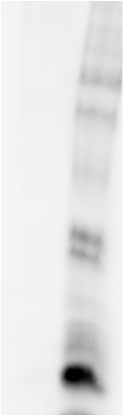

Fig. 2A RssR left panel

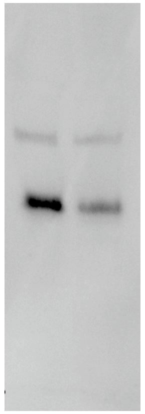

Fig. 2B STnc1740 left panel

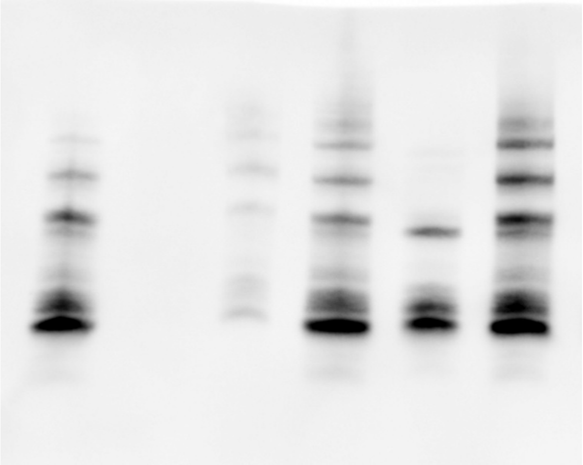

Fig. 2A RssR right panel

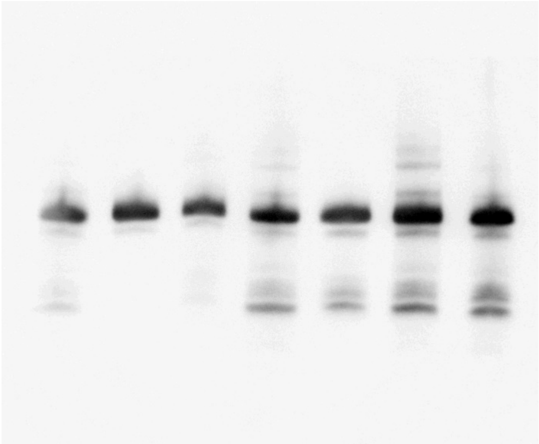

Fig. 2A 5S loading control right panel

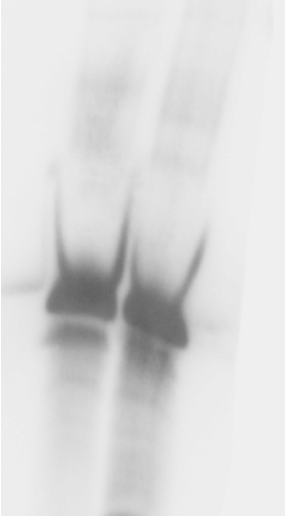

Fig. 2A 5S loading control left panel

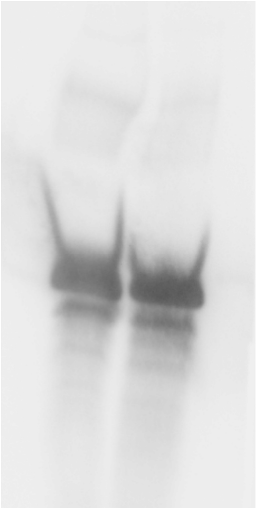

Fig. 2B 5S loading control left panel

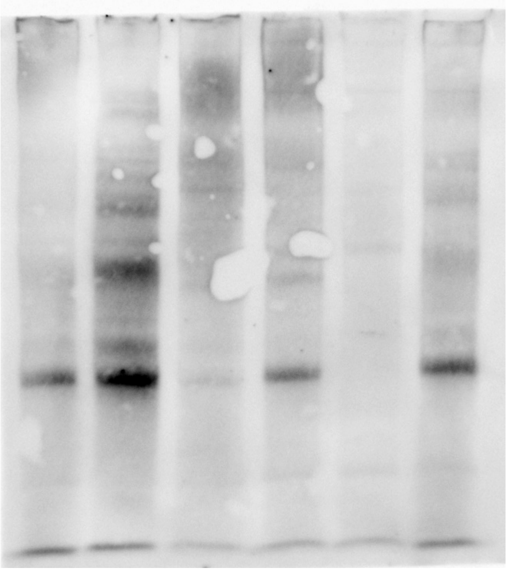

Fig. 2B STnc1740 right panel

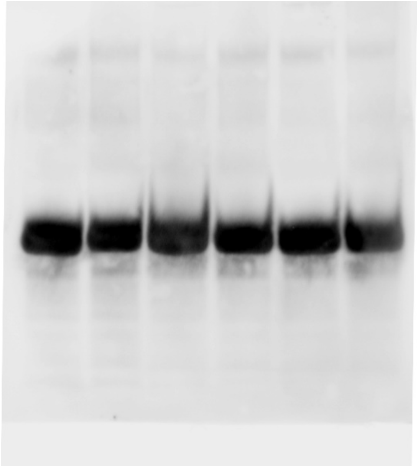

Fig. 2B 5S loading control right panel
